# Pediatric non-alcoholic Wernicke’s encephalopathy: a case report

**DOI:** 10.1186/s13052-025-02022-7

**Published:** 2025-07-21

**Authors:** Lucia Corso, Laura Lucaccioni, Martina Buttera, Mattia De Agostini, Patrizia Bergonzini, Elisabetta Spezia, Elisa Caramaschi, Diego Biondini, Viviana Durante, Lorenzo Iughetti, Barbara Predieri

**Affiliations:** 1 Postgraduate School of Paediatrics, University of Modena and Reggio Emilia, Modena, Italy; 2 Paediatric Unit, Department of Medical and Surgical Sciences of Mothers, Children and Adults, University of Modena and Reggio Emilia, Modena, Italy; 3 Surgical Paediatric Unit, Department of Medical and Surgical Sciences of Mothers, Children and Adults, University Hospital of Modena, Modena, Italy

**Keywords:** Wernicke encephalopathy, Paediatric, Thiamine deficiency, Nystagmus, Gallstone, Case report

## Abstract

Wernicke-Korsakoff syndrome is a neurological disorder caused by thiamine (vitamin B1) deficiency. This encephalopathy is typically suspected in alcoholics adults, but it is important to remember that other less known and suspected causes can determine the development of the non-alcoholic Wernicke’s encephalopathy. In non-alcoholic patients, the primary causes of Wernicke’s encephalopathy include hyperemesis gravidarum, restrictive diets and malnutrition, cancer, post-operative complications following bariatric surgery. Few data are reported regarding non-alcoholic thiamine deficiency, especially within the paediatric population. We describe the case of an 11-year-old Caucasian male with obesity who experienced prolonged emesis after the beginning of a strictly hypocaloric dietary regimen. This resulted in biliary colic episodes and subsequent necessity for cholecystectomy. The day after surgery, the patient developed acute visual impairment, horizontal nystagmus and diplopia, which were attributed to thiamine deficiency. Wernicke’s encephalopathy was suspected, so a blood sample was immediately collected to assay thiamine levels and empiric thiamine supplementation was started. Already from the day after the beginning of the treatment, the patient showed a significant improvement in his clinical conditions. This case study delineates clinical presentation, diagnosis, and treatment of our patient and provides information regarding the red-flag risk factors of non-alcoholic Wernicke’s encephalopathy in children. The aim is to increase the likelihood of suspecting the diagnosis and to promptly start the therapy, which is both simple and lifesaving.

## Background

Wernicke’s encephalopathy (WE) is the acute phase of Wernicke-Korsakoff syndrome (WKS) and is characterised by a triad of eye movement disorders (external ophthalmoplegia and/or nystagmus), ataxia, and altered mental status. Nevertheless, only approximately 16–20% of patients manifest the complete triad. ¹ Wernicke’s encephalopathy arises from a nutritional deficiency of vitamin B1, an essential coenzyme for numerous pathways of the nervous system. The prognosis for affected patients is poor without an immediate treatment and the estimated mortality rate in adults is approximately 17%.^2^

WKS most frequently manifests in patients with a history of chronic self-neglect and alcohol use disorder, although it can also occur in other conditions associated with thiamine deficiency [3, 4]. Over the past decade, reports on non-alcoholic WKS slowly increased, leading to the identification of new groups of at-risk patients, mainly those experiencing vomiting or chronic diarrhoea, hyperemesis gravidarum, bariatric surgery’ post-operative complications, and inflammatory bowel disease [2, 5]. According to the literature, vomiting and weight loss are the primary factors responsible for the onset of non-alcoholic WKS [3]. 

In clinical practice, it is common to consider WE as an adult condition, associating its manifestation primarily with alcohol abuse. This approach has the potential to mislead clinicians and delay the diagnosis of non-alcoholic WE, particularly in paediatric patients [6, 7]. 

We report a case of Wernicke’s encephalopathy in an 11-year-old Caucasian obese male who experienced prolonged emesis after the beginning of a strictly hypocaloric dietary regimen. The prevalence of obesity and the use of strict dietary regimens among adolescents are on the rise, underscoring the necessity for paediatricians to be vigilant on potential life-threatening consequences of malnutrition due to an imbalanced nutritional intake.

Our aim is to illustrate the clinical presentation of WE in paediatric patients and to highlight the importance of raising awareness among clinicians that this condition can also affect children. As in adults, a prompt diagnosis is of the utmost importance in children and the early beginning of treatment is vital decreasing the risk of neurological complications.

## Case presentation

An 11-year-old Caucasian male with a severe obesity [(BMI 49.8 kg/m², + 2.9 standard deviation scores (SDS) according to the World Health Organization (WHO) growth charts] was admitted to the Emergency Department of a spoke hospital because of nausea, persistent vomiting, and right upper quadrant pain. Over the previous two months, he had lost 33 kg following a restrictive diet without multi-vitamin supplementation. Before the onset of these symptoms, he was in good health and no hospitalisation was needed.

During the first week of hospitalisation, intravenous hydration, proton pump inhibitors, and ondansetron were administered, without any improvement of patient’s clinical condition. He was then referred to our hub hospital for surgical evaluation in suspected acute cholecystitis. Upon admission, the patient exhibited mild brownish vomiting (10 times per day), persistent right hypochondrium pain [assessed using the Visual Analogue Scale (VAS), with a score of 4], and feeding difficulties, no fever was detected. Murphy’s sign was minimally feasible and predictive due to the patient’s severe obesity. Blood test results demonstrated a mild coagulation prolongation (PT INR 1.39, APTT Ratio 1.13; normal range 0.80–1.20), a mild hyperbilirubinemia (1.20 mg/dL; normal range 0.16–1.10), hypertransaminasemia (GPT-ALT 72 U/L; normal range 10–40), hypokalemia (K 2.9 mEq/L), and moderate leukocytosis (13.000 WBC/µL, normal range 5.000–11.000 WBC/µL), inflammatory markers were slightly elevated (PCR 1.7 mg/dl; normal range 0.5-1.0 mg/dl). Both the vitamin K and the potassium supplementations were started together with ursodeoxycholic acid (600 mg/day in three divided doses), intravenous hydration, ibuprofen, omeprazole, scopolamine, and ceftazidime therapy. The abdominal X-rays did not reveal air-fluid levels or perforation, and the abdominal ultrasound showed a distended gallbladder with thickened walls and hyperechoic biliary debris, as well as hepatomegaly and steatosis. The paediatric surgical team advised that an elective cholecystectomy should be performed once the inflammation was resolved.

During the first seven-day of hospitalization in our hub hospital, the therapy we have set up allowed a slight clinical improvement. However, the patient continued to experience episodes of vomiting (approximately three to four episodes per day) and feeding difficulties. Subsequently, he developed a mild headache associated to higher-than-normal blood pressure (systolic pressure over 95°th percentile). This prompted the introduction of an ACE inhibitor therapy (2.5 mg per day) that led to a partial improvement of the patient’s headache. An echocardiogram ruled out the presence of structural heart disease. A second abdominal ultrasound was performed, identifying the presence of multiple gallstones and a pericholecystic hypoechoic area.

**Fig. 1:**
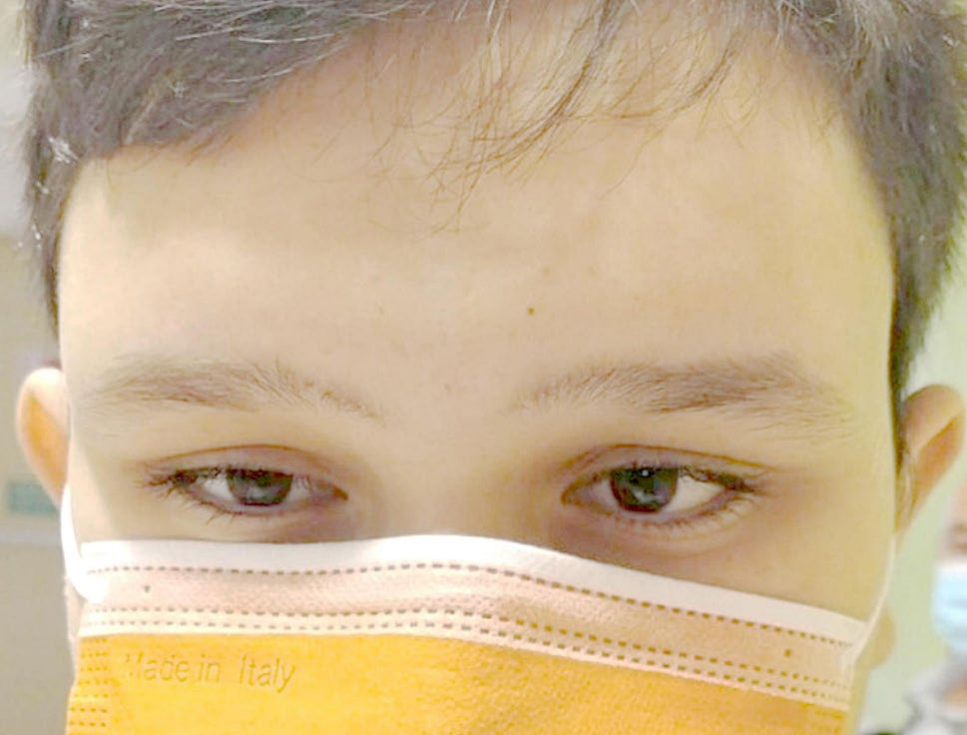
Convergent strabismus at onset

**Fig. 2:**
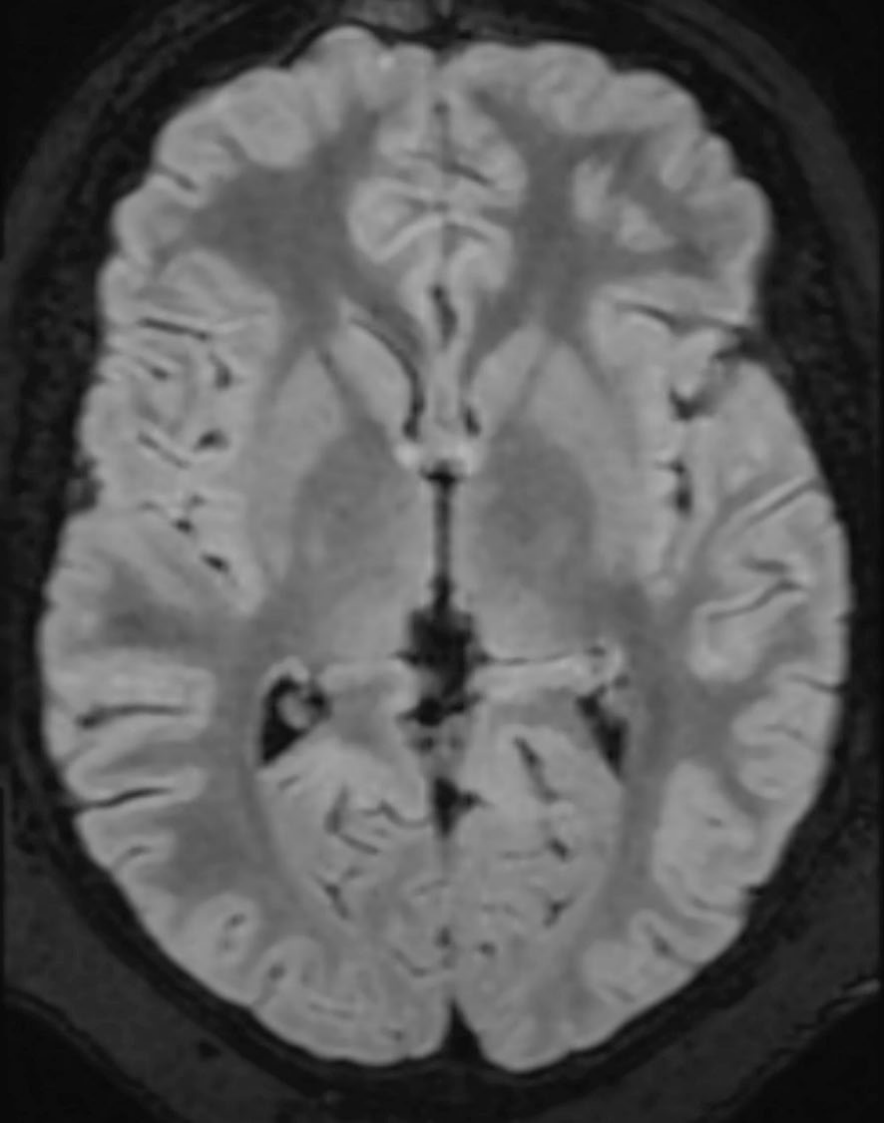
Axial MRI section at the thalamic-hypothalamic level in the 3D FLAIR sequence

On day 14, the patient underwent laparoscopic cholecystectomy without complications. After the surgical procedure, the patient’s vomiting stopped. However, within 24 h, he developed acute convergent strabismus (Fig. 1), binocular diplopia, bilateral horizontal gaze paralysis, left monocular vision impairment, and nystagmus during extreme gaze, in the absence of changes in the state of consciousness. An urgent brain CT scan excluded thromboembolic events, while the MRI scan revealed slight hyperintensity in the bilateral hypothalamic region, which was evident only in the 3D FLAIR sequence, reported as a possible normal finding, with no other notable abnormalities (Fig. 2). The electroencephalogram displayed no abnormalities. During the prolonged walk to assist the patient with the aforementioned tests, a widening of the gait and a newly unsteady gait were observed.Considering the patient’s history of prolonged fasting, significant weight loss, and vomiting, a diagnosis of Wernicke’s encephalopathy was considered. A blood sample was taken for thiamine assay, and parenteral thiamine therapy (500 mg three times per day for three days) was initiated in accordance with the recommendations set forth in the literature [1]. Symptoms improved significantly within 12 h, with resolution of strabismus and diplopia; however, fine nystagmus persisted. Additionally, a slight widening of the gait remained, though this improved over the subsequent four-day period. The patient was discharged in a well condition on a balanced diet and oral thiamine supplementation (300 mg/day). Before discharge, we collected a blood sample to reassess thiamine levels.

By the next follow-up examination, 10 days after discharge, the nystagmus resolved, and the thiamine supplementation was gradually reduced. Meanwhile, the result of the initial thiamine assay was received, indicating a vitamin B1 deficiency (18 ng/ml, normal range: 32–95 ng/ml) and supporting our diagnosis. Some days after, the analysis of the pre-discharge blood sample revealed normal thiamine levels.Oral thiamine (30 mg/twice a day) was continued for an additional month, after which the patient was managed by the hospital’s nutritional service.

**Fig. 3:**
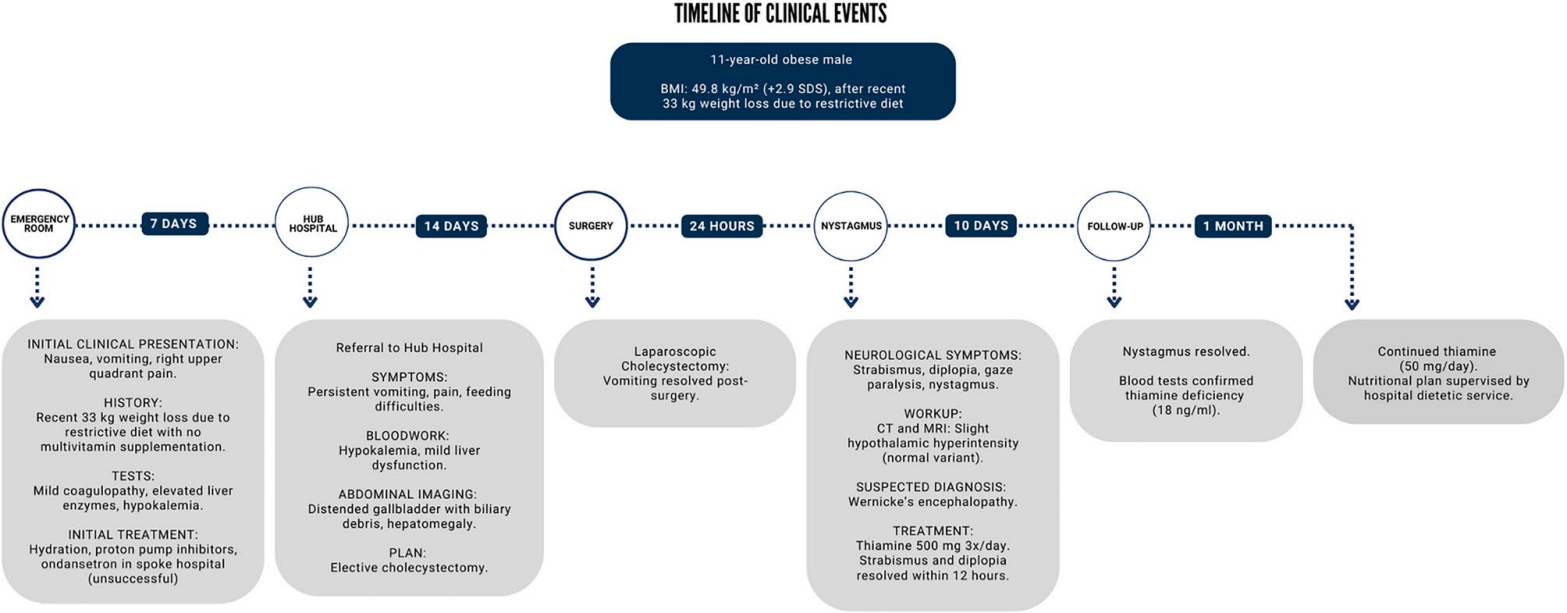
Timeline of clinical events

A summary timeline of the events pertaining to this clinical case is provided in Fig. 3.

## Discussion

Wernicke’s encephalopathy is less frequently observed in non-alcoholic patients. In adults, it is primarily reported as a complication following bariatric surgery (especially sleeve gastrectomy), during prolonged hyperemesis gravidarum in pregnancy, and in cancer patients [8, 9].

In the paediatric population, several cases of WE were described. Cases history remarkably similar to that of our patient were reported, involving severe weight loss, vomiting, and abdominal surgery [10–13].  A review published in 2016 reported nine cases of WE in children who had undergone weight loss surgery [14]. Furthermore, a systematic analysis of 586 cases of non-alcoholic Wernicke’s encephalopathy identified 33 children who developed the condition after consuming a soft drink diet and nine infants affected due to thiamine-deficient infant formula [3]. This study also observed that non-alcoholic Wernicke’s encephalopathy patients tend to be younger, with a mean age of 32.3 years. Table 1 provides a summary of paediatric WE cases, detailing the clinical presentation, primary causes, and the diagnostic usefulness of neuroimaging.

**Table 1:** Diagnosis number of cases age primary etiology symptomatology and MRI sensitivity of non-alcoholic Wernicke-Korsakoff in children

Diagnosis	Number of cases	Average age in years	Primary etiology and onset	Ataxia	Eye-movement disorder	Mental status change	Full Triad	MRI Sensitivity
Soft drink diet in babies and children [6]	33	1.3	22/33 vomiting, 11/33 infections, 7/33 diarrhea, 20 /33 lethargy, 15/33 edema	5/33	10/33	21/33	4/33	15/25
Accidental thiamine deficient infant formula [7]	9	0.5	8/9 vomiting, 9/9 infections, 7/9 lethargy, 5/9 irritability	0/9	3/9	9/9	0/9	1/2
Adolescent underwent Bariatric Surgery [13]	10	from 13 to 21	3/10 vomiting, 4/10 underwent anastomotic stenosis, 1/10 poor nutrient intake	4/7	4/7	5/7	2/7	Not Available

Modified from references 3 and 12

The increasing prevalence of obesity and the inclination to adopt online dietary regimens, frequently lacking validation by healthcare professionals, are considerable challenges. It is imperative that paediatricians can identify clinical signs of vitamin deficiencies, particularly thiamine deficiency, to facilitate prompt treatment and prevent serious neurological consequences [15]. The present case illustrates the vital importance of integrating suitable supplementation into the diets of obese children and ensuring that such regimens are overseen by duly qualified professionals. In this case the restrictive and unbalanced diet causing an important loss weight in a short period of time is the probable cause of gallstone formation, vomiting and subsequent thiamine deficiency.

The brain MRI (Magnetic Resonance Imaging) findings in our patient, namely “slight hyperintensity in the bilateral hypothalamic region evident in the 3D FLAIR sequence”, were mild and compatible with a normal variant. However, in both paediatric and adult patients, WE is frequently associated with symmetric hyperintensities in the mammillary bodies, periaqueductal grey, cerebellar vermis and thalamus on MRI [3, 16]. Previous studies indicated a correlation between brain MRI findings and the clinical severity of WE [17]. For instance, patients who are comatose often exhibit bilateral thalamic damage, whereas those who are not comatose typically show abnormalities in the periaqueductal grey matter. While MRI can support a WE diagnosis, it is important to note that up to 53% of patients with proven WE had normal brain MRI findings.

While MRI and thiamine levels facilitate the diagnosis of WE, it remains a clinical diagnosis, mainly in patients with a history of prolonged malnutrition. Chronic or recurrent vomiting may be an early indicator of thiamine deficiency, necessitating prompt assessment for timely diagnosis and prevention [18]. The optimal dosage of thiamine for WE prevention and treatment is still a topic of debate. In the context of prophylaxis, some studies proposed the daily administration of 250 mg of parenteral thiamine for a period of three to five days, particularly in patients who undergone bariatric surgery [19]. About confirmed cases of WE, the recommended therapy is the administration of 500 mg of parenteral thiamine three times a day for a period of three to five days [1]. When clinical improvement is observed, the patient should be administered 250 mg of IV thiamine daily for a period of 3 to 5 days, or until further improvement is no longer evident [1, 20]. Subsequent oral supplementation typically involves 30 mg twice daily for a minimum of one month. Our patient responded favourably to this treatment protocol, achieving complete clinical recovery.

## Conclusion

Non-alcoholic Wernicke-Korsakoff encephalopathy is a rare condition in children. However, the existence of documented cases underscores the necessity for heightened awareness and suspicion of this condition, even among paediatricians.

In the presence of red-flag risk factors for Wernicke’s encephalopathy, such as persistent prolonged vomiting, intentional or unintentional weight loss, malnutrition or even minimally invasive gastrointestinal surgery, children should be treated empirically as a potentially atypical case of WE. A delay in diagnosis and treatment may result in significant morbidity, irreversible neurological damage or even death. The treatment is straightforward and has the potential to prevent the development of an amnestic state associated with Korsakoff’s disease, which is a significant and potentially irreversible complication.

## Data Availability

Data sharing is not applicable to this article as no datasets were generated or analysed during the current study.

## Abbreviations

WKS: Wernicke-Korsakoff Syndrome
WE: Wernicke’s Encephalopathy
BMI: Body Mass Index
SDS: Standard Deviation Scores
WHO: World Health Organization
VAS: Visual Analogue Scale
MRI: Magnetic Resonance Imaging
